# *“I decided in my heart I have to complete the sessions”*: A qualitative study on the acceptability of an evidence-based HIV risk reduction intervention among women engaged in sex work in Uganda

**DOI:** 10.1371/journal.pone.0280138

**Published:** 2023-01-12

**Authors:** Ozge Sensoy Bahar, Proscovia Nabunya, Josephine Nabayinda, Susan Witte, Joshua Kiyingi, Edward Nsubuga, Simone Schriger, Jennifer Nattabi, Larissa Jennings Mayo-Wilson, Janet Nakigudde, Yesim Tozan, Fred M. Ssewamala

**Affiliations:** 1 Brown School, Washington University in St. Louis, St. Louis, MO, United States of America; 2 International Center for Child Health and Development, Brown School, Washington University in St. Louis, St. Louis, MO, United States of America; 3 Columbia University School of Social Work, New York City, NY, United States of America; 4 International Center for Child Health and Development Field Office, Masaka, Uganda; 5 Department of Psychology, University of Pennsylvania, Philadelphia, PA, United States of America; 6 Gillings School of Global Public Health, University of North Carolina, Chapel Hill, NC, United States of America; 7 College of Health Sciences, Makerere University, Kampala, Uganda; 8 School of Global Public Health, New York University, New York City, NY, United States of America; Nnamdi Azikiwe University, NIGERIA

## Abstract

**Background:**

The HIV burden remains a critical public health concern and women engaged in sex work [WESW] are at significantly higher risk compared to the general adult population. Similar to other sub-Saharan African countries, Uganda reports high rates of HIV prevalence among WESW. Yet, they have not been targeted by theory-informed HIV prevention intervention approaches.

**Methods:**

We conducted semi-structured in-depth interviews with 20 WESW upon intervention completion to explore their experiences with an evidence-based HIV risk reduction intervention that was implemented as part of a combination intervention tested in a clinical trial in Uganda (2018–2023. Specifically, we explored their initial motivations and concerns for participating in the intervention, barriers and facilitators to attendance, and their feedback on specific intervention characteristics.

**Results:**

The main expectations revolved around access to health-related information, including information on STIs, HIV, and PrEP as well as on how one can protect themselves while engaging in sex work. Initial concerns were around potential breach of confidentiality and fear of arrest. The main facilitators for session attendance were the motivation to learn health-related information, the attitude of facilitators, and the incentives received for participation, whereas main challenges were related to family commitments and work schedules. WESW appreciated the group format of the intervention and found the location and times of the intervention delivery acceptable.

**Discussion and conclusions:**

Overall, our findings suggest that the HIV risk reduction intervention was appropriate and acceptable to WESW. Yet, WESW experience unique concerns and barriers that need to be accounted for when designing interventions targeting this population, especially in resource-limited settings where sex work is illegal and highly stigmatized.

**Clinical trial registration:**

NCT03583541.

## Introduction

Women engaged in sex work (WESW) bear the greatest burden of HIV globally [1, 2]. In 2019, female sex workers had a 30 times greater risk of acquiring HIV than the general female population [2]. In Uganda, the HIV prevalence among sex workers is estimated at 31% compared to the national average of 5.4% (15–49 years) [3]. Even with advances and expansion in HIV care and treatment, WESW across sub-Saharan Africa (SSA) continue to have suboptimal HIV prevention and treatment outcomes [4].

The unmet HIV prevention and treatment needs within sex work significantly contribute to overall HIV transmission [5], partly due to challenges associated with coverage of effective HIV prevention and treatment services within this population [6]. More specifically, the criminalization of sex work and punitive legal environments increase vulnerabilities among WESW and further push them into unregulated and unsafe working environments, which increases the risk of engaging in high-risk behaviors, and relatedly their risk for HIV acquisition and transmission [7, 8]. These factors combined with poverty, gender-based violence, high mobility, substance use, and intersecting HIV stigma, including at health facilities, limit access to and uptake of HIV prevention and treatment services among WESW [9, 10].

While strategies to expand HIV prevention and treatment services could have a considerable impact on the spread of HIV among WESW, the effectiveness of these strategies depend on the extent to which sex workers engage in these programs. Studies have examined the barriers and facilitators for engaging in HIV care and treatment services, including HIV testing and counseling, linkage to HIV care, and treatment adherence [4, 11–13]. Among WESW, awareness of the existence and importance of services, attitudes toward services, and HIV risk perception determine access and utilization of health services [11, 14, 15]. Indeed, women who perceive themselves to be at a higher risk of HIV are more likely to uptake and continue with services [16]. Additionally, stigma may discourage women from accessing services for fear of stigmatization, discrimination, social exclusion and loss of customers or work termination [11, 14, 15, 17].

Location and adequacy of facilities providing health care also impact access to care. Mixed findings have been reported with some women expressing a preference for closer facilities to save time and transportation [18]. Other women prefer far facilities away from their residence due to perceived lack of privacy, breach of confidentiality, and stigmatization at health facilities [14, 18]. Additionally, facility working hours determine whether women access and uptake health services or not [10, 19].

Affordability of services, including fees for services, transport, and other costs also determine access and use of health services. Scorgie and colleagues found that women were four times more likely to go for services if they were offered at a low cost or free [10]. However, even for free services, transportation costs and long wait times may become barriers to service uptake [10]. Finally, the appropriateness of services in meeting the needs and expectations of women, especially on the first encounter with health providers influences the continuation of services. Specifically, stigma and discrimination and poor attitudes of health providers, including judgmental treatment, name-calling, and delaying or denying services [17]. These attitudes, combined with unfriendly service protocols, including preconditions for accessing services, such as requiring identity cards, further deter women from accessing care [10, 14, 19].

While there are studies examining barriers and facilitators to WESW’s access and use of health services related to HIV testing and counseling, and HIV treatment, there is limited research on the acceptability and appropriateness of comprehensive HIV prevention interventions that specifically target WESW, apart from studies that examine the acceptability of specific HIV risk reduction strategies, such as PrEP or condom use among this population [20–23]. Also, few studies reported on the feasibility and acceptability of interventions targeting WESW prior to implementation [24, 25] or examined service provider perceptions of feasibility and acceptability of implementing evidence-based practices for preventing HIV/AIDS and STIs among WESW [26]. In Uganda, studies among WESW have explored knowledge, attitudes, barriers and facilitators to HIV prevention and treatment services, as well as preference of service delivery models [27–30]. One study examined WESW’ perceptions of HIV self-testing and PrEP use following the intervention [31, 32]. Yet, WESW’s perspectives on evidence-based comprehensive behavioral HIV risk reduction interventions specifically targeting this population are understudied.

In this article, we examine WESW’s perspectives on the acceptability and appropriateness of the HIV risk reduction component of a combination intervention delivered in communities in southwest Uganda as these perspectives are critical in ensuring the sustainment and scalability of these interventions.

## Methods

The larger study, funded by the National Institute of Mental Health is a randomized clinical trial (2018–2023) that evaluates the efficacy of adding economic empowerment components to traditional HIV risk reduction intervention to reduce new incidences of STIs and of HIV among WESW in HIV hotspots (sites) in the greater Masaka region of Uganda. Please see study protocol for more details [33]. Study sites were randomly assigned to three conditions: 1) Control arm receiving HIV risk reduction sessions focused on equipping participating women with the skills to reduce the spread of HIV; 2) Treatment arm 1 receiving HIV risk reduction sessions combined with a matched savings account and financial literacy training with integrated behavioral economics principles, aimed at training participants on issues related to the importance of savings, banking services, budgeting, and debt management; and 3) Treatment arm 2 receiving HIV risk reduction sessions, a matched savings account, financial literacy training, and vocational skills training and mentorship sessions, aimed at economically empowering women for purposes of starting up an income-generating activity. Treatment arms 1 and 2 were combined under Treatment arm 1 due to COVID-19 that interfered with recruitment and data collection.

Women were eligible to participate if they met the following conditions: 1) 18+ years; 2) reported having engaged in vaginal or anal intercourse in exchange for money, alcohol, or other goods in the past 30 days; and 3) reported engagement in one or more episodes of unprotected sex in the past 30 days (see study protocol for further details, [33]).

The study used an embedded experimental mixed methods design [34] where qualitative data was collected post-intervention across all three arms at 6-month (Time 1), 12-month (Time 2), and 24-month (Time 3) follow-up to be collected).

### Study setting

The study is conducted in the Greater Masaka region, consisting of 7 political districts. The average HIV prevalence among 15–49-year-olds across the 7 districts was estimated at 9.5%, above the national average of 5.4% (Uganda AIDS Commission, 2021). HIV prevalence among WESW in Rakai and Masaka districts was reported as high as 61% [35].

### Study intervention

The study tested a combination intervention that comprised of: 1) HIV risk reduction sessions; 2) Financial literacy training; and 3) Matched savings individual development account. The study findings reported here focus on the acceptability of the HIV risk reduction sessions. Hence, only the HIV risk reduction intervention is described here (see study protocol for details on the other study interventions).

### HIV risk reduction intervention

**Fig 1:**
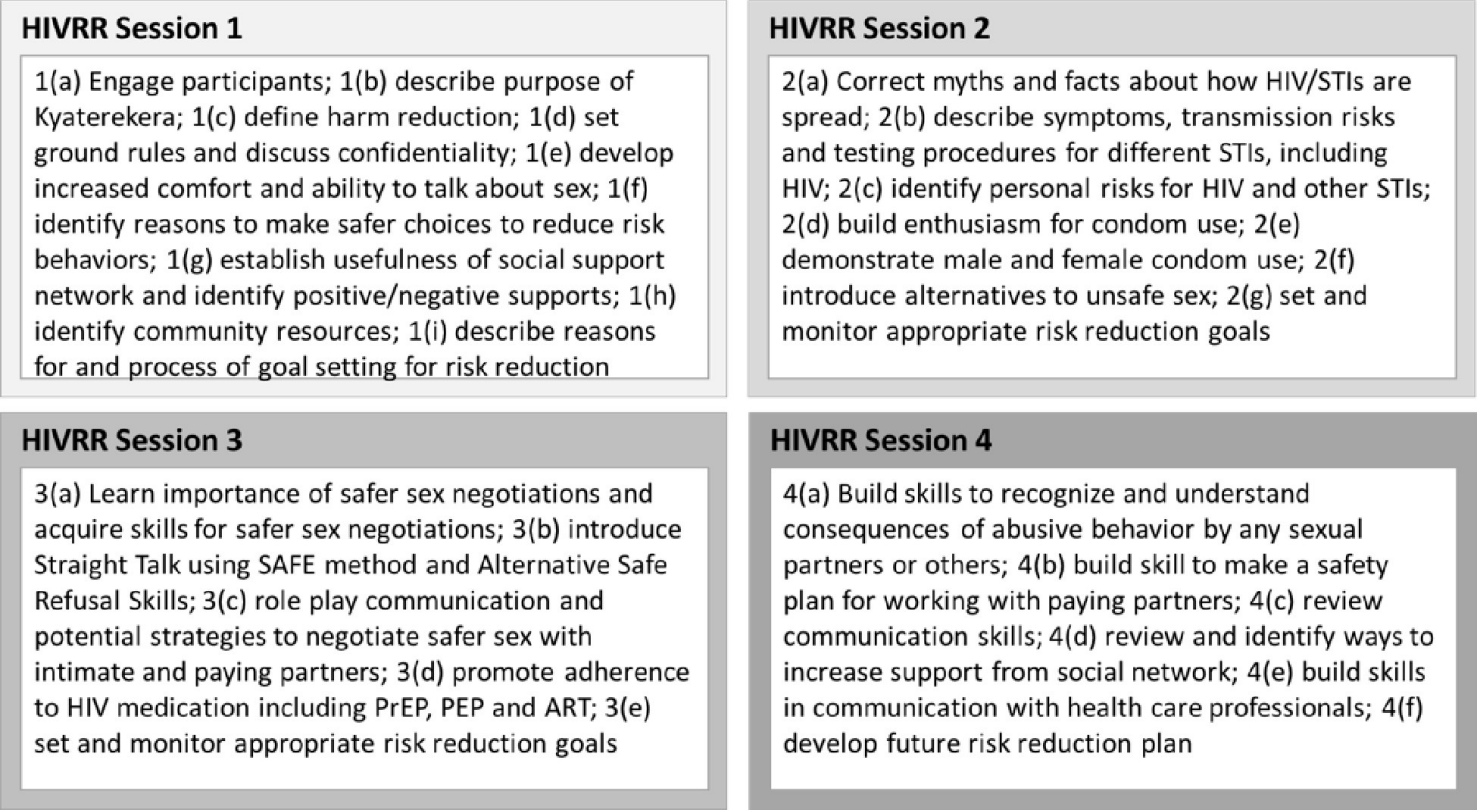
HIVRR session content [33].

Guided by social cognitive theory [36, 37] and a harm reduction approach [38, 39], the HIV risk reduction component of the Kyaterekera study focused on both sexual and drug/alcohol use risk reduction, and is designed to increase communication, problem-solving skills, and self-efficacy related to safe-sex behaviors and substance use (see Fig 1. HIVRR session content). The intervention incorporated many of the social cognitive theory’s social-cognitive mediators within the HIV prevention context, including male and female condom use, communication skills, and building social support. The harm reduction approach is a well-accepted pragmatic approach to high-risk behaviors if abstinence from the behavior is not achievable [38, 39] and is infused throughout the curriculum.

All women in the study received four sessions of the evidence-based, HIV/STI risk reduction intervention tested in three previous studies conducted in Central Asia [40, 41]. The sessions were facilitated by community health workers who participated in a one-week training conducted by one of the study principal investigators and a study consultant with expertise in HIV risk reduction interventions. The 2-hour sessions were delivered twice a week during weekdays for two weeks in groups of 20 to 25 WESW. Sites with one group received the sessions in the morning. At sites with more than one group, the sessions were delivered in the morning and afternoon. WESW received transport refund and lunch at each session that they attended. The attendance rate across four sessions for the whole sample (n = 542) was 72 percent.

### Qualitative sample selection

A stratified purposive sampling strategy was used to represent the wide range of experiences [42]. The qualitative sampling was based on participants’ attendance of intervention sessions: HIV risk reduction (4 sessions) and financial literacy training (6 sessions). For each of the 19 sites, one participant from the highest, mid (average), and lowest quartiles of the total number of attended sessions was selected [n = 57]. Due to intervention delivery delays related to COVID-19, for Time 1 qualitative data collection, semi-structured interviews were completed with only 20 participants from seven sites across 3 districts. Fifteen participants were from the control arm of the study and five from the combined treatment arm.

### Qualitative data collection

In this study, we report findings from semi-structured in-depth interviews conducted following intervention completion (Time 1) with 20 participants. Data from 17 participants were collected in December 2019. The remaining three interviews were completed in December 2020 given the delays in intervention completion due to COVID-19. The Time 1 interviews focused on: 1) participants’ experiences with the respective intervention and its specific components (i.e., HIVRR, savings, and financial literacy); 2) key multi-level (individual, economic, family, contextual, and programmatic) influences that affected their participation; and 3) perceptions on intervention sustainability. Interview questions used for this study included, but were not limited to: *“Could you tell us a little bit about why you decided to participate in the program*?*”*, *“What parts of the project*, *if any*, *made you hesitate to take part*?*”*, *“Can you tell us about your experience attending the sessions*?*”*, *“What were the things/factors that made it easier for you to attend the sessions*?*” “What were the factors that made it difficult for you to attend the sessions*?*”* and “W*hat did you think of the way the sessions were delivered*?*”* Probing questions were added where relevant. The full interview guide for Time 1 has been published elsewhere [43].

Interviews were conducted in Luganda, the widely spoken language in the study region. Interview questions were translated (English to Luganda) and back-translated by two proficient team members. Questions were reviewed and revised by the study team that included native speakers to ensure that they sounded natural and conversational, and conveyed the intended meanings. Interviews were conducted by trained research assistants fluent in both Luganda and English and lasted 38 to 100 minutes (mean = 60 minutes). All interviews were conducted in a private place with only the research assistant and the participant present and were audiotaped.

### Qualitative data analysis

Interviews were first transcribed verbatim and then translated from Luganda to English by research assistants fluent in both languages. Dedoose analytic software was used for data analysis. We used Proctor et al.’s conceptualization of implementation outcomes to guide our analysis [44]. Specifically, we focused on the concepts of acceptability and appropriateness from the consumers’ perspectives–in this case, WESW. Acceptability is defined as the perception that a given treatment, service, practice, or innovation is agreeable, palatable, or satisfactory. Acceptability of a particular intervention or a set of interventions is assessed based on the stakeholder’s knowledge of or direct experience with various dimensions of the implemented intervention, such as its content, complexity, or comfort. Appropriateness refers to the perceived fit, relevance, or compatibility of the intervention, including for the target population.

We used inductive techniques for thematic analysis of the data [45–47]. Thematic analysis allowed us to use preexisting themes, such as barriers and facilitators to participation but also derived subthemes that emerged from the data, such as “sex trafficking” and “concern for confidentiality of information”. Interview transcripts were initially read multiple times and independently coded by three authors using sensitizing concepts informed by the existing literature and the content of the intervention as well as identifying emergent themes (open coding) [48]. Broader themes were broken down into smaller, more specific units until no further subcategory was necessary. Initial codes were discussed during team meetings and reorganized when necessary to create a final codebook that was used to code all transcripts.

The secondary analysis, conducted by two authors, compared and contrasted themes and categories to identify similarities, differences, and relationships among findings. Peer debriefing and audit trail were used to ensure rigor [49, 50]. The codes and the findings were presented to two members of the research team who were not involved in the data analysis to discuss the plausibility of themes and related findings [49].

#### Ethical considerations

Study procedures were approved by the Washington University in St. Louis Institutional Review Board (#201811106), Columbia University Institutional Review Board (IRB-AAAR9804) and the in-country local Institutional Review Boards in Uganda: Uganda Virus Research Institute (UVRI–GC/127/18/10/690), and the Uganda National Council of Science and Technology (UNCST–SS4828). Written consent was obtained from all WESW in the study. The study is also overseen by an in-country Data Safety and Monitoring Board, as detailed in the study protocol [33].

## Results

### Participant demographics

Participants’ age ranged between 20 to 46 years (mean age: 31.45). Seven participants were sampled in the low attendance, six in average attendance, and seven in high attendance groups from across seven sites. Thirty percent of the participants were married or in a relationship, 90% had primary school education and 10% had secondary education. Fifty percent tested HIV positive and 15% tested positive for any of the STIs (Chlamydia, Gonorrhea, and Trichomonas). The average age at sex work debut was about 24 and the average number of sex partners within the last 30 days was 32 (see Table 1. Participant demographics per study arm). To maintain participant privacy and confidentiality, pseudonyms are used throughout.

**Table 1:** Participant demographics per study arm.

Variable	Control (N = 15) (n)	Treatment (N = 5) (n)	Total Sample (N = 20) (n)
Age (Mean, SD) (min/max = 20–46)	30.87 (6.03)	33.20 (5.36)	31.45 (5.83)
**Marital status**			
Married	2	1	3
Divorced	8	3	11
Separated	1	0	1
Widowed	1	0	1
In a relationship	2	1	3
Single, never married	1	0	1
**Highest level of education**			
Did not go to school	3	1	4
Dropped out before primary 7	8	0	8
Completed primary 7 and stopped	2	0	2
Dropped out before senior 4	1	3	4
Completed senior 4 and stopped	1	1	2
**Number of people in the household** (min/max = 1–6)	2.60	2.60	2.60
**Age when first did sex work** (min/max = 14–35)	22.40	28.60	23.95
**Number of years in sex work** (min/max = 1–23)	8.40	4.60	7.45
**Number of sex partners in the past 30 days** (min/max = 2–120)	24.20	54.40	31.75
**HIV Status**			
Positive	9	1	10
Negative	6	4	10
**STI Status**			
Positive	1	2	3
Negative	14	3	17
**HIVRR attendance per session**			
Session 1	14	4	18
Session 2	8	5	13
Session 3	9	3	12
Session 4	12	3	15

### Program acceptability and appropriateness

To capture the full picture in terms of acceptability and appropriateness of the intervention, we explored WESW’s expectations and concerns about the program prior to enrolment as well as the barriers and facilitators to their participation after enrolment. We also inquired into their thoughts regarding the different characteristics of the intervention, including group format, day and time of the sessions, and session facilitators.

#### Initial expectations from the kyaterekera program

We asked WESW what they expected to get from the Kyaterekera program when they first heard about it. The main expectations revolved around access to health-related information, including information on STIs, HIV, and PrEP as well as on how one can protect themselves while engaging in sex work. For instance, Gloria, engaged in sex work for about 15 years, wanted to learn about alternative ways of reducing risks associated with unprotected sex.

*Gloria*: *I expected to learn how to minimize the risks brought with engaging in sex with men without protection*, *which may lead to acquiring of diseases*.

Barbara, who started engaging in sex work 10 years ago, was motivated by what the program had to offer in terms of HIV testing and the possibility of being initiated on medication.

*Barbara*: *I expected a lot*. *That woman told me that*, *they give medicine*, *they can test*, *and you know your status; if you find out that you are sick (HIV positive)*, *you can be initiated on medication and if they find you healthy*, *you can protect yourself further*.

Ruth, engaged in sex work for four years, stated that she “expected little”. Her expectations were primarily focused on receiving “protective gear” and learning how to use them. She went on to share that the program exceeded her expectations.

*Ruth*: *I did not expect much*. *(I expected that they would) Give us protective gear and teach us the best way to use them*. *I expected condoms and their access points as well as encouraging participants to stay safe…*. *I expected little*, *we learned a lot*.

Esther, engaged in sex work for about 15 years, was particularly interested in learning about PrEP, including how to take the pills as well as the limits of its protection against HIV.

*Esther*: *(I expected to learn about) Taking (PrEP) pills*, *having sex with a man who is not wearing (condom)*, *and using a man who is drunk*…. *I expected to learn how to take pills*, *that once you take them*, *you prevent yourself from acquiring the disease*, *but also if you have sex with a man who hasn’t taken the pills*, *yet you have taken (them)*.

Finally, Sanyu, who had been in sex work for over five years, pointed out that she was motivated by the possibility of change for a healthier life hoping “*to change my ways*….*we were using a lot of intoxicating substances and drugs”*.

#### Initial concerns about participating in the kyaterekera program

We also asked WESW what made them hesitate to participate when they first heard about the Kyaterekera program. While some (about one-third) said they were not concerned about anything, others expressed various concerns that reflected the unique vulnerabilities WESW experience as they navigate their relationships outside their trusted network, including when services are made available. The most cited concern related to program participation was a breach of confidentiality, mentioned by about one-third of our sample. This included being broadcast on TV or on the internet. Anitah, engaged in sex work for more than five years, pointed out the way they were already treated by other people and was particularly concerned about their children learning that she was engaged in sex work. She was then reassured by the research team that her information would be kept confidential.

*Anitah*: *Given the insults we get from other people with some labeling us as prostitutes*, *I feared being aired on TV for everyone to see including my children*…. *Later*, *I was assured of my safety and that everything was confidential*.

Other WESW also expressed concern that their information would be disclosed on the internet or broadcast on television. In addition to concerns around disclosure on the internet, Alice, engaged in sex work for over 10 years, was also concerned that the information she gave could be used to arrest her. Her worries were eased by the research team. She added that the team *“remained true to your word”*.

*Alice*: *I thought that they intend to share this information on the internet or arrest us*, *however*, *I asked about my concern and the research team assured me that the information will be treated with the utmost confidentiality*, *and this calmed me down*.

Being arrested was a fear shared by other WESW as well, especially given that sex work is illegal in Uganda. For Afiya who had been engaged in sex work for the last couple of years, her worry was exacerbated by the fact that WESW were asked to bring their national identification cards at the time of enrollment for citizenship verification as WESW from other nationalities also worked in the area where the study was conducted.

*Afiya*: *I expected them to arrest us so as we were going there*, *we felt suspicious because we had been told that we had to go with our national identification cards*, *yet the sessions were health-related*. *This made me terrified*. *When I reached there*, *I first didn’t give out my identification card until they explained to me well about the program*.

Gloria, who has been engaged in sex work for about 15 years, was also worried about being arrested, but “*she (the nurse) explained to me that the project aimed at seeing how the female sex workers can live sustainably*.” Ultimately, she was motivated by both the “*hope*” that she would be tested for STIs and by the “*interest of learning how best to protect myself*” and attended all HIV risk reduction sessions.

Though mentioned by only two WESW, being taken “*abroad to have sex with animals”* was a concern. These narratives reflected the stories that WESW heard in their work where women were being trafficked abroad and forced to engage in sex with animals, as was also discussed during our community collaborative board meeting.

Afiya shared that she was also worried about being killed. Although she was the only one to raise this concern, her narrative points out the harsh realities sex work may entail in Uganda.

*Afiya*: *Since they first selected some of us before informing our other colleagues*, *we asked ourselves why they had first informed us*. *I got worried because many of our colleagues had been killed after someone told them that something was going on in a particular place and when they went there*, *the next thing you heard was their death*!

#### Facilitators to participation in HIVRR sessions

One of the major reasons why WESW kept attending the sessions was the “desire to learn more,” pointing to the high relevance of the session contents to the lives of WESW. Almost all WESW mentioned that they came back to the sessions because they wanted to obtain more information on a range of issues, including information on HIV and STIs, reducing risk behaviors, and *“handling clients”*. For instance, Ruth mentioned that she learned new things in each session *“at zero cost”*.

*Ruth*: *You could think that you know yet you are ignorant*. *Although I knew something about HIV*, *I wanted to learn more and indeed a lot was covered in the sessions*. *With every session introduced*, *new things were taught to us that I did not know*. *This compelled me to continue coming with hopes of learning new ideas at zero cost*.

In addition to learning about how to protect herself, especially about condom use, Gertrude, engaged in sex work for five years, was also motivated to come back by the *“inspirational”* messages she heard from facilitators during the sessions. When asked to elaborate, she shared:

*Gertrude*: *Even your inspirational words that you used to give us about protecting ourselves were motivating us to come back for subsequent sessions*…. *such as we should not leave condoms for more money because a man might force you*, *we should stop using drugs*, *reduce them or stop using them completely so that we can do things when we are conscious about*, *not for the implications of unprotected sex getting to use the condom*.

Kirabo, who had been engaged in sex work for about 10 years, wanted to learn more about how to reduce risk-taking behaviors, including drug and alcohol use and number of sexual partners. Kirabo went on to add that she reduced her drug and alcohol use as well as the number of clients as a result of attending the HIVRR sessions.

*Kirabo*: *I attended these sessions such that I reduce or quit drug use and alcohol or even reduce the number of sexual partners*. *That was the only reason for my coming*…. *I found out that if you always drink alcohol*, *marijuana*, *use “amayirungi” (khat)*, *and shisha*, *you may end up doing inappropriate things because you are high on these*. *I learned that when one reduces drug use*, *they might end up making informed decisions*. *I use that information well*. *I used to take many types of drugs but now I stopped taking some of the drugs and also reduced the number of sexual partners*

A few participants also mentioned that in addition to the content, the way the facilitators and research staff related to them made it easier for them to continue attending the sessions. Alice, Ruth, as well as Flora engaged in sex work for six years, discussed how they appreciated the facilitators’ attitude, care, and attention, underlining the importance of facilitators in engaging and retaining participants, especially among highly stigmatized groups. Ruth talked about how she was treated by the HIVRR session facilitators.

*Ruth*: *Most of the time people despise women engaged in sex work yet we are also humans like them*. *The facilitators respected us and taught us whole-heartedly*, *they did not speak ill about us nor did they abuse us*. *I was so happy about that*.

Another common reason mentioned by about three-quarters of the WESW was the incentives offered. WESW received monetary compensation for their time and transport and were offered lunch after each session they attended. Additionally, free condoms were made available to those who wanted them. Free condoms were mentioned by four WESW. Joyce, who recently started sex work, stated that she was “*driven by two factors*: *teaching us about our health and giving us condoms”* and acknowledged that sometimes she attended the sessions to receive free condoms.

Half of the WESW in the sample appreciated receiving transport refunds and about one-third receiving food. For instance, Maria, who had been engaging in sex work for five years, pointed out that in addition to learning, what motivated her to attend sessions were the “*compensation and food”*. Afiya stated, *“Sometimes we used to leave our work and come for the sessions because we needed the money”*. Anitah shared that the transport refund made it easier for her to get transport back home and motivated her to attend subsequent sessions: “I used to get a transport refund after every session that I attended. This made it easier for me to get transport means back home. Therefore, it motivated me to attend subsequent sessions.”

Sanyu first appreciated the content she learned during the sessions, but also acknowledged being compensated for their time and transport played a role in her participation since she and her colleagues were sometimes “*too poor*”.

*Sanyu*: *Another thing was the incentives; sometimes we were too poor and when we received that small incentive*, *we were motivated to go back for the sessions expecting to receive that allowance*.

#### Challenges to participation in HIVRR sessions

When asked about what made it challenging for them to participate in the HIVRR sessions, about one-third of the WESW mentioned family commitments. These included taking care of other family members or loss of relatives. For instance, Gertrude missed sessions to be able to attend to her sick mother who was taken to the hospital. Esther was only able to attend one session because she lost a relative and was away during the remaining three sessions. Viola, engaged in sex work for about five years, talked about her husband’s expectation from her to be home when he arrived, which conflicted with the timing of the sessions.

*Viola*: *At times*, *it would be hard for me to keep to the set time for the sessions because my husband usually comes home during that time*. *He expects me to be home by that time*, *yet I also want to attend the session*.

Anitah had children in her care and sometimes her commitments to her children conflicted with the timing of the sessions, making it challenging for her. However, she was able to come up with a solution that allowed her to both attend the sessions and make sure her children had food.

*Anitah*: *I had to prepare food for the children before they returned from school*. *At times*, *I failed to prepare food in time because sessions had taken longer than expected* …*I would use part of the transport refund to buy cooked food for the children after the session had ended*.

About one-fifth of the WESW also discussed how their work and work schedules posed a challenge. Flora stated that sometimes work could conflict with the sessions, but she was able to work around her work schedule to make sure she attended all sessions as she *“decided in my heart and said I have to complete the sessions*.” She also chose to attend to her other chores after the sessions. Grace, engaged in sex work for about five years, also talked about her work, specifically alluding to the *“nature of our job”*. She mentioned that the long work hours at night would leave her tired and sleep deprived.

*Grace*: *The nature of our job would sometimes make it a challenge for us to come and attend the sessions*. *Imagine you worked at night*, *stayed awake all night*, *you were on the street and then you got very little money*, *you are sleepy*.

Alice had another business that occasionally made it difficult for her to attend the sessions, especially when sessions ran long or started later than the scheduled time.

Other challenges included personal reasons. For instance, Betty, engaged in sex work for a couple of years, and Sanyu were nervous about the results of their HIV testing that was done before the intervention started as they were worried about their HIV status. Both were relieved when they found out they were negative and attended the HIVRR sessions.

Ruth and Barbara were concerned about their privacy. Barbara was worried that the other WESW she was attending the sessions with were calling her Neko (woman engaged in sex work) in front of her friends. Ruth was worried about being seen coming to sessions at first, given the *“nature of my work”*, yet she was reassured when she saw other WESW attend the sessions.

*Ruth*: *At first*, *I found it difficult*, *I feared that people would know the nature of my work when they see me coming for these sessions*…. *I decided*, *to attend the sessions without any fear because I saw my colleagues who used to come directly to the venue without hiding*. *This gave me the courage*.

Grace was in jail at the time HIVRR sessions started. Once she was out, she made sure to attend all other sessions. Other personal reasons included being *“drunk”* and being in *“low mood”*, each mentioned by one WESW. In both cases, WESW shared that they still managed to attend the sessions.

#### Intervention characteristics

*Group format*. The eight WESW who responded to this question all shared positive feedback about the group format. They appreciated being with a group of people where everybody *“who went there were engaged in sex work*.*”* This allowed them to share their experiences without fear of being judged and learn from others who share similar experiences. For instance, Flora shared that being among WESW brought a level of comfort because they *“could ask any questions knowing that everybody that was in the group was doing the same job as we were*.*”*

Barbara was encouraged to make changes to her alcohol consumption. She felt like she was not alone when she heard her other colleagues expressing similar challenges.

*Barbara*: *One of them was saying*, *“according to the way I was drinking alcohol*, *my alcohol consumption has now reduced” because when you drink alcohol and get drunk*, *any person can take you*. *Another one said*, *“I used to consume 10 bottles of alcohol but now I reduced to 5 bottles”*. *That was what attracted me most because I also used to drink alcohol but when I started attending the sessions*, *I realized a gradual change in my life*.

For Afiya, the benefits of being in a group with other WESW were two-fold, one that she was with people with similar experiences and two that whatever she said would not be repeated outside the group. She was further reassured by the facilitators’ emphasis on confidentiality.

*Afiya*: *I got a feeling that I wasn’t alone*, *and that other people were facing challenges like mine*. *This built intrinsic courage within me*. *The other thing was that they emphasized confidentiality by telling us not to share the experiences contributed during the sessions with any other people*. *It helped me a lot when I knew that we were all engaged in sex work*, *I relaxed because it meant that my secrets would not leak but stay within our circle*.

Maria appreciated being in a small group and getting a chance to know everyone. She also appreciated the rules that were put in place to ensure that groups were run smoothly.

*Maria*: *We also used to follow the rules which were written on the charts like not talking during sessions and listening and respecting each other’s opinions*…. *raising a hand if you want to say something because if you talk anyhow*, *you may create confusion*.

*Delivery location*. The locations for the sessions were identified in collaboration with the site coordinators at each site who manage WESW (including managers, brothel owners, pimps, lodge owners). WESW were content with the settings where the sessions were conducted. They appreciated that the sessions were held in convenient, quiet, and “isolated” places that allowed them to maintain their privacy. Given the nature of their job, privacy was particularly important to WESW. Grace shared that she liked the venue because *“it was secluded and not everyone would see you and start saying that what are these sex workers doing there*…*”* Joyce shared similar thoughts and liked that the place was private, but not too isolated to draw other people’s attention as to what one might be doing there.

*Joyce*: *The place was also isolated*. *It was near to this community*, *and no one could ask you where you were coming from*. *They could easily think that you were going to the market*, *yet you were branching off to this place*. *It was also private; passersby couldn’t easily see us*.

WESW also talked about the quietness of the setting as an important feature as they were able to focus on the content and not get distracted. Betty thought that a quiet place was “*a conducive environment to communicate without any interruption”*. Finally, convenience was also appreciated by a few WESW. Joyce mentioned that the place where the sessions were held was near, which made it easier to attend as she was able to walk when she did not have transport money. Mariam, engaged in sex work for over 10 years, also shared that the place *“could be easily located and accessed”* by WESW.

Two WESW had concerns about the setting where the sessions were delivered. These concerns were related to privacy. For instance, Esther stated that most women, including herself, did not like the place where the sessions were held because “*some of them are married*, *so they are doing sex work in hiding"* and the setting was not private enough. Patience, engaged in sex work for four years, also shared that they had to switch settings for the sessions and the current place was less private as *“some people came and peeped through the windows and asked what we were doing*.*”*

*Day and time of the sessions*. The day and time of the sessions were scheduled in consultation with the WESW. Hence, WESW were content with the day and time of the sessions, which were scheduled on the weekdays and in the morning at sites with smaller groups, and morning and afternoon at sites that had a larger number of WESW. WESW appreciated the morning hours as those were the times when they did not have any clients. Some expressed that they would prefer to meet a little later in the day, around noon, so that they would have time to rest or attend to other chores they might have after working through the night. WESW also appreciated the reminder calls from the research team. For instance, Ruth shared that the reminder calls allowed her to plan accordingly and added that people’s attendance depended on how invested they were.

*Ruth*: *(I wasn’t inconvenienced) because they would call you a day before and inform you about the time and day then you prepare yourself accordingly*. *Besides*, *we were not blamed for missing a session*. *So*, *it was up to you*, *with the interest you have in the program*.

Ruth went on to add that if it were up to her, she would prefer the weekends, especially Sundays when she could attend the sessions after church. Viola and Kirabo also stated that they would have scheduled the sessions on the weekends as they found it more convenient.

*Facilitators*. WESW appreciated the facilitators delivering the HIVRR sessions. They found the information and the way it was conveyed by the facilitators helpful. Anitah shared that the sessions were covered in detail and questions were encouraged. She added that the facilitators *“gave us enough time during the sessions; they used to review the session for those that came in late such that we all move at the same pace*.*”* Other WESW stated that facilitators used visual aids such as demonstrations and photos, which made it easier for them to understand the content as illustrated by Joyce:

*Joyce*: *What was helpful about it was bringing visual aids which we used to learn from*. *It would be hard for us to understand what they were teaching us without them*. *We used visual aids and learned practically instead of just using words*.

WESW were appreciative of being treated with respect and patience. Gertrude shared that the facilitators were always helpful in answering their questions and offering clarification.

*Gertrude*: *Even the questions we used to ask for what we had not understood*, *or for any areas where we wanted more clarity so we would ask the facilitator and she would give you the answer which saved you when what you thought was wrong or even giving you more knowledge on that*.

Both Ruth and Ritah engaged in sex work for over 20 years, referred to being treated *“humanely”*. Ruth went on to add that this enabled the group to share their thoughts and experiences without any reservation.

## Discussion

In this study, we explored the acceptability of the evidence-based HIV risk reduction component of a combination intervention that also included an economic empowerment component among WESW in Uganda. We explored the expectations and concerns that contributed to their decisions to participate in the overall program, facilitators, and barriers to their participation in the HIV risk reduction sessions, as well as thoughts on different aspects of intervention delivery. WESW’s expectations mainly centered around access to health-related information and resources as well as information around safe sex practices, which shows that the program was highly relevant and appropriate to the needs of this population. For those who expressed concerns about participation -about two-thirds of our sample, these potential benefits ultimately outweighed their initial concerns around enrolment to the program. However, concerns around confidentiality -including disclosure of their engagement in sex work, being arrested, killed, or trafficked for sex work underline the unique vulnerabilities of WESW. These concerns were also identified in other studies as barriers to access to services and underline the need to effectively address them to facilitate access to interventions as well as other health services designed for this population [14, 17, 18, 51].

In addition to being the initial reason for enrolment in the program, the desire to learn more information on HIV/STIs and safer sex practices was the main reason that motivated WESW to continue attending HIV risk reduction sessions. As expected, when WESW find the intervention relevant and applicable to their life circumstances, they are more likely to sustain their participation. Importantly, in addition to content relevance, some WESW also pointed to the fact that being treated respectfully by the facilitators was another factor that facilitated their continued attendance. The same theme emerged when WESW were asked about their feedback on intervention implementation. Stigma WESW experience from service providers is a major factor that interferes with access and retention with health services, including HIV care [14, 17, 18, 51].

In addition to not being stigmatized, WESW appreciated that facilitators were patient and took the time to answer questions, underlining the importance of training facilitators/service providers working with this key population to improve WESW’s engagement and retention in services. As indicated before, the HIVRR facilitators delivering this intervention participated in a one-week training that included sensitization around working with WESW in addition to intervention content and delivery. Finally, WESW also stated that monetary compensation for their time and transport, as well as lunches, made it easier for them to attend the sessions. Costs associated with transportation are widely cited as a barrier to attendance [10, 52]. By providing transport refund, we aimed at minimizing barriers to WESW’s attendance at the sessions. However, doing so has cost implications for the implementation of the intervention in the real world and its scalability.

Barriers to HIVRR session participation included family commitments, work schedules, and other personal reasons which primarily centered around privacy and confidentiality. Many women were also caregivers to children or other members of their families. One participant experienced the loss of an extended family member that interfered with her session attendance. In addition, even though the research team worked closely with WESW and their managers to identify the most convenient times for intervention delivery, work schedules still posed challenges for some WESW. Both family and work commitments are common barriers to participation across a wide range of programs and services [53–55]. Hence, our findings show that WESW experience some of the same barriers as the general population to accessing and utilizing services [53].

However, there were also challenges unique to the life circumstances of WESW, though mentioned by only a few, including being intoxicated or in jail. Concerns around privacy and confidentiality remained a concern for some WESW during the initial sessions, underlying the need to continuously reinforce the message that all information would be kept confidential as well as put in place safeguards to ensure it. While ensuring privacy and confidentiality is critical in any group intervention, doing so is even more critical for WESW given the high level of stigma, discrimination, and other risks associated with disclosure of sex work [11, 14, 15, 17].

Overall, WESW’s feedback on intervention characteristics was positive. WESW who provided feedback on the group format found it helpful. Being in a group with other WESW not only made them more comfortable to share their experiences, but some WESW stated that it also encouraged some to make changes to their risk-taking behaviors. These findings align with the existing evidence on the benefits of group interventions. The group format for behavioral interventions in the context of HIV prevention utilizes the social interactions among group members to enhance the likelihood of acquiring new information and skills, facilitate behavioral change, and enable the group members to learn with and from each other in a safe environment [56].

Except for a few instances, WESW also expressed satisfaction with the locations where the intervention was delivered, emphasizing their convenience and privacy. Similarly, WESW were also generally satisfied with the day and time when the sessions were delivered. Both the delivery location and timing of the sessions were decided upon in collaboration with WESW and their managers in an attempt to minimize potential obstacles to attendance. As discussed before, WESW’s feedback on facilitators delivering the intervention was also positive and emphasized the attention, care, and respect they conveyed during the sessions.

Overall, our findings suggest that the HIV risk reduction component of our combination intervention was appropriate and acceptable to WESW. From a public health point of view, our findings show that WESW experience unique concerns and barriers to enroll and attend interventions that need to be addressed to increase the acceptability of an intervention. Privacy and confidentiality are one such concern that warrants the attention of service providers and/or program implementers working with WESW. Safeguards to ensure privacy and confidentiality should be incorporated both at the time of enrolment as well as throughout the intervention, including the selection of sites where it will be implemented. In addition, interventions targeting WESW should incorporate both content and sensitivity training for facilitators/service providers to improve access and utilization [57]. Finally, working closely with WESW and their managers in the identification of appropriate locations and times may increase the acceptability of an intervention. Alvarez identified the use of group formats and co-creating interventions with the target population [58], in addition to the use of theoretical frameworks to inform a program/intervention -in our case social cognitive theory, as three program characteristics being particularly effective. From a policy perspective, our findings align with existing evidence that proposes the enactment of policies to create a safe and favorable environment for WESW to access and attend programs and services without fear of being arrested, prosecuted, or discriminated against [59].

Results need to be interpreted in light of study limitations. Due to restrictions related to COVID-19 at the time of qualitative data collection, we collected data from a smaller sample of WESW than originally targeted. Relatedly, WESW in the treatment group were underrepresented. Hence, the findings may not fully represent the experiences of WESW who received the combination intervention.

Despite these limitations, our study provides important insights into the acceptability of an evidence-based HIV risk reduction intervention among WESW in hot spots in Uganda–a region underserved by evidence-based interventions. Our study is one of the few studies in Uganda, and in SSA, that documents the unique needs and challenges of WESW from their own perspectives when HIV risk reduction programs are provided.

## Conclusion

Overall, our findings suggest that WESW found the HIV risk reduction intervention acceptable and were satisfied with their experiences. Privacy and confidentiality were among the initial concerns and remained as such for the first couple of sessions, underlying the importance of effectively implementing safeguards and conveying them to WESW to ensure their enrolment and utilization. Our findings also highlight the importance of providing sensitization training to facilitators/service providers delivering the intervention. Finally, deciding on the delivery location and time in collaboration with WESW and their managers, when possible, may minimize some of the barriers to access and utilization of services and/or interventions provided. These findings have important programmatic and policy implications in Uganda. The Uganda Government and Ministry of Health identify sex workers as a key population. This population is one of the groups targeted by combination HIV prevention, care and treatment, and social support interventions, as outlined in the National Strategic Plan for HIV and AIDS [60]. As such, given that WESW are a high-risk population for acquiring and transmitting STIs, including HIV, it is important to consider incorporating evidence-based interventions with high acceptability into the national programs and policy efforts and that take into account the unique needs of WESW to address HIV in Uganda.

## Supporting information

S1 Table Codebook developed for data analysis. (DOCX)
