# Supplementary material for: “I decided in my heart I have to complete the sessions”: A qualitative study on the acceptability of an evidence-based HIV risk reduction intervention among women engaged in sex work in Uganda
Source: PLoS One. 2023 Jan 12;18(1):e0280138. doi: 10.1371/journal.pone.0280138 (PMC9836279; doi:10.1371/journal.pone.0280138)
Supplement: S1 Table — (DOCX) [file pone.0280138.s001.docx]

| **Themes** | **Subthemes** |
| --- | --- |
| Initial program expectations | - Access to health-related information - *-on STIs* - *-PrEP* - *-protected sex* - HIV testing & linkage to care - “Change for the better” |
| Initial concerns | - Breach of confidentiality - Fear of arrest - Trafficking for forced sex with animals - Fear of being killed - No concerns |
| Facilitators to attendance | - Desire to learn more information   - on STIs and HIV   - on reducing risk behavior   - “handling” clients - Treatment by staff and facilitators - Incentives   - Transport refund   - Lunch   - Free condoms |
| Challenges | - Family commitments   - Care of family members   - Family loss - Work schedules   - Sex work   - Other - Personal reasons   - Concern about HIV status   - Concern about privacy   - Being in jail   - Being drunk   - Being in “low mood” |
| Intervention characteristics |  |
| - Group format | - Shared experience - Encouragement to share - Reassurance for confidentiality - Small group format to know everyone |
| - Delivery location | - Convenience - Quietness - Privacy |
| - Day and time of delivery | - Convenience with work schedule - Reminder calls |
| - Facilitators delivering the sessions | - Content covered in detail - Use of visual details - Respect - Patience |

**S1 Table. Codebook developed for data analysis**
